# Multi-ancestry Mendelian randomization of omics traits revealing drug targets of COVID-19 severity

**DOI:** 10.1016/j.ebiom.2022.104112

**Published:** 2022-06-27

**Authors:** Jie Zheng, Yuemiao Zhang, Huiling Zhao, Yi Liu, Denis Baird, Mohd Anisul Karim, Maya Ghoussaini, Jeremy Schwartzentruber, Ian Dunham, Benjamin Elsworth, Katherine Roberts, Hannah Compton, Felix Miller-Molloy, Xingzi Liu, Lin Wang, Hong Zhang, George Davey Smith, Tom R. Gaunt

**Affiliations:** a Department of Endocrine and Metabolic Diseases, Shanghai Institute of Endocrine and Metabolic Diseases, Ruijin Hospital, Shanghai Jiao Tong University School of Medicine, Shanghai, China; b Shanghai National Clinical Research Center for metabolic Diseases, Key Laboratory for Endocrine and Metabolic Diseases of the National Health Commission of the PR China, Shanghai Key Laboratory for Endocrine Tumor, State Key Laboratory of Medical Genomics, Ruijin Hospital, Shanghai Jiao Tong University School of Medicine, Shanghai, China; c MRC Integrative Epidemiology Unit (IEU), Bristol Medical School, University of Bristol, Oakfield House, Oakfield Grove, Bristol, BS8 2BN, United Kingdom; d Renal Division, Peking University First Hospital, Peking University Institute of Nephrology, Key Laboratory of Renal Disease, Ministry of Health of China, Key Laboratory of Chronic Kidney Disease Prevention and Treatment (Peking University), Ministry of Education, Beijing, People's Republic of China; e Wellcome Sanger Institute, Wellcome Genome Campus, Hinxton, Cambridgeshire CB10 1SA, United Kingdom; f Open Targets, Wellcome Genome Campus, Hinxton, Cambridgeshire CB10 1SD, United Kingdom; g European Molecular Biology Laboratory, European Bioinformatics Institute (EMBL-EBI), Wellcome Genome Campus, Hinxton, Cambridgeshire CB10 1SD, United Kingdom; h Bristol Medical School, University of Bristol, 5 Tyndall Avenue, Bristol, BS8 1UD, United Kingdom; i Department of Microbiology and Infectious Disease Centre, School of Basic Medical Sciences, Peking University Health Science Centre, Beijing, China; j NIHR Biomedical Research Centre at the University Hospitals Bristol NHS Foundation Trust and the University of Bristol, United Kingdom

**Keywords:** Drug targets, COVID-19 severity, Multi-ancestry, Mendelian randomization, Colocalization

## Abstract

**Background:**

Recent omic studies prioritised several drug targets associated with coronavirus disease 2019 (COVID-19) severity. However, little evidence was provided to systematically estimate the effect of drug targets on COVID-19 severity in multiple ancestries.

**Methods:**

In this study, we applied Mendelian randomization (MR) and colocalization approaches to understand the putative causal effects of 16,059 transcripts and 1608 proteins on COVID-19 severity in European and effects of 610 proteins on COVID-19 severity in African ancestry. We further integrated genetics, clinical and literature evidence to prioritise drug targets. Additional sensitivity analyses including multi-trait colocalization and phenome-wide MR were conducted to test for MR assumptions.

**Findings:**

MR and colocalization prioritized four protein targets, FCRL3, ICAM5, ENTPD5 and OAS1 that showed effect on COVID-19 severity in European ancestry. One protein target, SERPINA1 showed a stronger effect in African ancestry but much weaker effect in European ancestry (odds ratio [OR] in Africans=0.369, 95%CI=0.203 to 0.668, *P* = 9.96 × 10^−4^; OR in Europeans=1.021, 95%CI=0.901 to 1.157, *P* = 0.745), which suggested that increased level of SERPINA1 will reduce COVID-19 risk in African ancestry. One protein, ICAM1 showed suggestive effect on COVID-19 severity in both ancestries (OR in Europeans=1.152, 95%CI=1.063 to 1.249, *P* = 5.94 × 10^−4^; OR in Africans=1.481, 95%CI=1.008 to 2.176; *P* = 0.045). The OAS1, SERPINA1 and ICAM1 effects were replicated using updated COVID-19 severity data in the two ancestries respectively, where alternative splicing events in *OAS1* and *ICAM1* also showed marginal effects on COVID-19 severity in Europeans. The phenome-wide MR of the prioritised targets on 622 complex traits provided information on potential beneficial effects on other diseases and suggested little evidence of adverse effects on major complications.

**Interpretation:**

Our study identified six proteins as showing putative causal effects on COVID-19 severity. OAS1 and SERPINA1 were targets of existing drugs in trials as potential COVID-19 treatments. ICAM1, ICAM5 and FCRL3 are related to the immune system. Across the six targets, OAS1 has no reliable instrument in African ancestry; SERPINA1, FCRL3, ICAM5 and ENTPD5 showed a different level of putative causal evidence in European and African ancestries, which highlights the importance of more powerful ancestry-specific GWAS and value of multi-ancestry MR in informing the effects of drug targets on COVID-19 across different populations. This study provides a first step towards clinical investigation of beneficial and adverse effects of COVID-19 drug targets.

**Funding:**

No.

**Research in context**

**Evidence before this study**

We searched key terms in PubMed published before Feb 1st 2022, with the terms: (“COVID-19 or “coronavirus”) AND (“omics” or “protein” or “transcript”) AND (“Genome-wide association study” or “Mendelian randomization”). We found multiple studies identified genes or proteins associated with COVID-19. However, there is little human genetics evidence to identify whether the host genes/proteins associated with COVID-19 severity are ancestry-specific or consistent across ancestries.

**Added value of this study**

To our knowledge, this is the first comprehensive genetic study that identified protein targets that showed effect on COVID-19 severity in European and African ancestries. Our study identified one protein, SERPINA1, that showed effects on COVID-19 in African ancestry (OR=0.369, *P* = 9.96 × 10^−4^), but weaker in European ancestry (OR=1.021, *P* = 0.745). This implies that increased SERPINA1 levels may reduce COVID-19 risk in Africans. In addition, our study identified four additional protein targets, FCRL3, ICAM5, ENTPD5 and OAS1, that showed effect on COVID-19 severity in Europeans. FCRL3, ICAM5 and ENTPD5 showed weaker effects in African ancestry, where OAS1 had no reliable genetic predictors in Africans. One protein ICAM1 showed suggestive effect in both ancestries, where alternative splicing may be a mediator linking ICAM1 with COVID-19 severity.

**Implications of all the available evidence**

Our study prioritised six drug targets for COVID-19 severity, four of them showed different levels of evidence in European and African ancestries. Our study also highlights the value of ICAM1 in relation to COVID-19 severity in both ancestries. This is the very first step of drug target prioritization/de-prioritization for COVID-19 severity. Whether drugs of these targets showed similar effect needs further investigation in future clinical trials.

Alt-text: Unlabelled box

## Introduction

The outbreak of Severe Acute Respiratory Syndrome Coronavirus 2 (SARS-CoV-2), the causative agent of novel coronavirus disease 2019 (COVID-19), has been a global pandemic. It has infected a large proportion of the world's population with a wide spectrum of manifestations, ranging from completely asymptomatic carriers to critical respiratory failure, multi-organ dysfunction, and death.^1^ This heterogeneity may relate to different host responses involving multiple human proteins and pathways. Considering the relatively high mortality rate of patients with severe disease,^2^ search for optimal treatments, such as novel drugs to improve survival rate of severe patients is a key objective to reduce the impact of both the current and potential future coronavirus epidemics.

Currently, some clinical trials have shown beneficial effects of some approved drugs on COVID-19, including IL6R antagonist^3^ ACE inhibitor,^4^ Peginterferon Lambda-1a,^5^ fluvoxamine,^6^ Calcium release-activated calcium (CRAC) channel inhibitors,^7^ Janus kinase 1/2 inhibitor,^8^ vitamin D.^9^ In parallel, some experimental studies have identified a set of human proteins that interact with SARS-CoV-2,^10^ and may provide drug targets for general coronavirus interventions. Some genetic and epidemiology studies suggested that drug response of COVID-19 differs among ethnic groups. For example, Ammar et al showed that IL6R inhibition was likely to show more impaired response for COVID-19 in sub-Saharan Africans compared to East Asians and Europeans.^11^ Some cost-effective approaches, such as genetic tools, can be applied to investigate the effects of drug targets on COVID-19 across ancestries.

Omics data could play a role in supporting efficient drug development for various diseases including COVID-19. Recent studies have begun to support the role of genetics in predicting drug trial success.^12^ Some multi-omics studies have further demonstrated the value of molecular quantitative trait locus (QTL) studies in repurposing existing targets to additional indications as well as prioritizing novel drug targets.^13^ One approach to utilising molecular QTL data is Mendelian randomization (MR).^14^^,^^15^ MR uses genetic variants as instrumental variables to estimate the effect of an exposure (e.g. measured levels of a protein) on an outcome (e.g. COVID-19 severity), which could prioritise drug targets cost-effectively. Recent multi-ancestry genetic studies have further showcased the value of omics analyses in predicting treatment response in various populations.^16^^,^^17^

Recent GWAS^18^^,^^19^^,^^20^ and MR studies^21^, ^22^, ^23^ utilising data from the COVID-19 Host Genetics Initiative (HGI; https://www.covid19hg.org/) and GenOMICC consortium^19^^,^^24^ have identified a set of genes (e.g. *ABO, OAS1, IFNAR2, IL10RB*) associated with various COVID-19 phenotypes. One recent genetic study utilized large-scale omics traits to understand the role of the SARS-CoV-2 receptor ACE2 on COVID-19 and other complex diseases.^25^ However, there are issues that require consideration: i) some genes and proteins are known to be pleiotropic, which may violate the “no pleiotropy” (or “exclusion restriction”) assumption of MR; ii) when comparing severe COVID-19 cases with population controls as an outcome, it is not possible to separate the causal effects of becoming infected from any causal effects on disease progression after infection (despite these potentially being separate mechanisms);^26^ iii) collider bias (also known as selection bias, sampling bias or ascertainment bias).^27^ Each of these could induce spurious associations between the target and COVID-19. Careful instrument and outcome selection are needed for the drug target MR of COVID-19.

The aim of this study was to prioritise potential drug targets for COVID-19 severity as well as to identify potential beneficial and adverse effects of these targets on other diseases. We combined genetic association information on 16,059 transcripts and 1608 proteins in European ancestry^13^^,^^28^^,^^29^ and 610 proteins in African ancestry,^16^ and applied a recent omics MR analysis pipeline^17^ to estimate the causal effects of these targets on COVID-19 severity in the two ancestries separately. To enable rapid queries, results of all analyses are available in an open access online platform (https://epigraphdb.org/covid-19/ctda/).

## Methods

### Study design and participants

**Figure 1:**
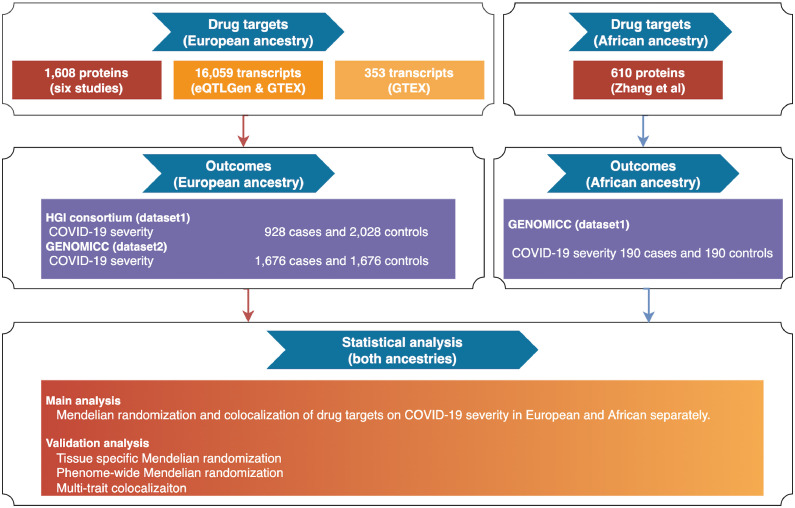
Study design of the multi-ancestry Mendelian randomization study to prioritise drug targets for COVID-19 severity.

Figure 1 describes the design of this study. We aimed to prioritise drug targets for COVID-19 severity in European and African ancestries. Three sets of exposures that proxy drug target effects have been implemented in this study: i) expression levels of 1608 proteins (N≤7212; Table S1A) and 16,059 genes (N≤31,684; Table S2) from European ancestry; ii) expression level of 610 proteins from African ancestry (N≤1871; Table S1B); iii) tissue-specific gene expression of 353 genes with literature evidence of association with COVID-19 (Table S3-5). The outcome was COVID-19 severity from two studies (HGI N cases=928, N controls=2028; GenOMICC Europeans N cases=1676, N controls=1676; GenOMICC Africans N cases=190, N controls=190; Table S6). The putative causal effects of the selected drug targets on COVID-19 severity were estimated using MR and colocalization in European and African ancestries separately. Several sensitivity analyses including multi-trait colocalization and phenome-wide MR were conducted to validate core MR assumptions.

### Instruments selection for drug targets

The circulating levels of proteins and expression levels of genes were selected as exposures in this study since they represent drug targets for many drugs. To generate genetic instruments for potential drug targets, genetic variants robustly associated with the levels of proteins and gene transcripts (with *P* < 5 × 10^−8^), irrespective of genomic position of variants, were selected from six proteome datasets extracted from two studies^13^^,^^16^ and two transcriptome datasets.^28^^,^^29^

As shown in Figure 1, three sets of genetic instruments that proxy drug target effects were selected in this study: i) 7092 instruments of 1608 plasma proteins and 39,630 instruments of 16,059 gene expressions in the European ancestry; ii) 3550 instruments of 610 proteins in the African ancestry; iii) 1493 instruments of 353 COVID-19 related drug targets from the literature (Table S5). All instruments that passed the FDR threshold of 0.05 in the original omics GWAS had F-statistics over 10 (which reflects sufficient instrument strength of these variants for MR analysis). All instruments are conditionally independent of each other. The eQTL and sQTL instruments were in almost no linkage disequilibrium (LD r^2^<0.001). For pQTL instruments, 272 of 5326 (5.1%) of the instruments in European ancestry and 54 of 3543 (1.5%) variants in African ancestry were in low to modest LD (r^2^ between 0.2 to 0.6; Table S1A and 1B). These pQTLs were obtained from Zhang *et al.,*^16^ which were conditional independent pQTL signals after conditional analysis and genetic fine mapping. More details of the instrument selection were described in Note S1.

### Outcome selection

The genetic associations with COVID-19 severity were obtained from the COVID-19 HGI (https://www.covid19hg.org/). Among the six available genetic association datasets listed in Table S6, we considered the hospitalized COVID-19 vs. not hospitalized COVID-19 as the most suitable dataset to represent COVID-19 severity. The major advantage of this data compared to other datasets (e.g. COVID-19 vs. population) is that all participants of this genetic analysis were confirmed hospitalized COVID-19 cases, differing only by severity of subsequent disease. Whilst population controls provide a potential reference for infection, there will be some asymptomatic individuals with SARS-COV-2 infection, making it less effective. A comparison of diagnosed COVID-19 cases and population controls would conflate exposure, susceptibility to infection and disease severity, preventing us distinguishing prevention and treatment targets. We therefore used the GWAS summary statistics of the hospitalized COVID-19 vs. not hospitalized COVID-19 as the outcome for the MR analysis. This GWAS combined European samples from three large scale biobanks: UK Biobank, DECODE and FinnGen (928 confirmed hospitalized COVID-19 patients, 2028 non-hospitalized COVID-19 patients), although asymptomatic testing information were not provided. To validate and identify additional causal genes and/or proteins, we used the COVID-19 severity dataset from the GENOMICC study.^19^ Both European- and African-specific datasets of COVID-19 severity were used in this study (Table S6A). The COVID-19 severity cases were defined as COVID-19 cases that required continuous cardiorespiratory monitoring or required hospital admission. The ancestry-matched controls were selected from the UK Biobank in a 1:1 ratio with the cases.

### Statistical analysis

#### Mendelian randomization analysis

In the main analysis, the effects of selected proteins and transcripts were tested against COVID-19 severity using MR in European (HGI data and GenOMICC data) and African ancestries (GENOMICC data) separately. The Wald ratio method was used to obtain MR effects for protein and/or transcript targets with only one instrument. For protein and/or transcript targets with two or more instruments, the generalized inverse variance weighted (IVW) method was used to estimate the MR effects, as the method takes into account the weak LD among instruments using a LD matrix.^30^ As a validate, we conducted MR for the prioritised proteins using data from HGI v6 and GenOMICC v2 (Table S6B).

#### Colocalization analysis

Genetically predicted associations between a protein or gene and COVID-19 severity may arise from any of four scenarios: causality, reverse causality, confounding by LD between the leading SNPs for proteins and phenotypes or horizontal pleiotropy (Figure S1). For drug targets that showed robust MR effects on COVID-19 severity in the main MR analysis, (Bonferroni corrected threshold, P for protein< 5.97 × 10^−5^ or P for transcript< 3.56 × 10^−6^), we conducted single-trait colocalization^31^ to distinguish causality from confounding by LD (using the “coloc” R package). This approach estimates the posterior probability of each genomic locus containing a single variant affecting both the target and the phenotype.^31^ We used the default prior probabilities that a variant is equally associated with each phenotype (p1=1 × 10^−4^; p2=1 × 10^−4^) and both phenotypes jointly (p12=1 × 10^−5^). For genomic regions with multiple QTL signals, we applied pair-wise conditional and colocalization method (PWCoCo),^13^ which relaxed the colocalization assumption of a single genetic signal in the test region by estimating the colocalization probability for each pair of genetic signals of a target-COVID-19 association. For a given target-COVID-19 association, only the PWCoCo result with the largest colocalization probability was presented to show the most informative finding. The PWCoCo result using marginal summary statistics was noted as “marginal”. The result using joint summary statistics conditional on the top hit was noted as “conditional”. A posterior probability above 70% for the colocalization hypothesis in these analyses would suggest that the two association signals are likely to colocalize within the test region (noted as “Colocalised”). The rest of the target-phenotype associations were noted as “Not colocalised”.

#### Tissue Specificity analysis

The functional receptor of SARS-CoV-2, ACE2, is highly expressed in multiple organs, including gastrointestinal tract, gallbladder, testis, and kidney. This is consistent with the likelihood that whilst SARS-CoV-2 infection primarily manifests with acute respiratory illness, the presence of SARS-CoV-2 in the alimentary tract for longer than in the respiratory system^32^ suggests that the intestine may be a hidden reservoir of SARS-CoV-2. We therefore set out to explore differences in potential target effects in different tissues. In order to understand the tissue-specific effects of the candidate targets of COVID-19 on human phenotypes, we selected the 9 tissues in which ACE2 is highly expressed, including testis, lung, kidney cortex, kidney glomerular, kidney tubulointerstitial, stomach, colon transverse, small intestine terminal ileum and colon sigmoid.

Tissue-specific gene expression data of the 353 targets in each selected tissue were obtained from GTEx V8.^29^ After selection, 580 instruments of 218 gene transcripts were selected in the 9 tissues, which included 141 instruments for 132 transcripts in testis, 125 instruments for 115 transcripts in lung, 20 instruments for 20 transcripts in kidney cortex, 6 instruments for 6 transcripts in kidney glomerular, 8 instruments for 8 transcripts in kidney tubulointerstitial, 71 instruments for 67 transcripts in stomach, 84 instruments for 81 transcripts in colon sigmoid, 98 instruments for 96 transcripts in colon transverse and 41 instruments for 39 transcripts in small intestine terminal ileum (Table S5). The same MR and colocalization analysis pipeline was applied for the tissue specific analysis.

#### Multi-trait colocalization analysis of prioritised targets

For protein and/or transcript targets with robust MR (P_protein< 5.97 × 10^−5^ or P_transcript< 3.56 × 10^−6^) and colocalization (probability >70%) evidence, we explored whether the causal variants were shared across transcriptome, proteome and COVID-19 severity. We applied multi-trait colocalization implemented in the moloc R package.^33^ The default prior probabilities of 1 × 10^−4^ for any one layer of association, 1 × 10^−6^ for any two layers of associations and 1 × 10^−7^ for colocalization of all three layers of associations were used in the moloc analysis. An overall colocalization probability of three traits (Pa,bc+Pab,c+Pac,b+Pabc) >70% would suggest that the three association signals are likely to colocalize within the test region.

#### MR PheWAS of prioritised targets for COVID-19 severity

For the 353 prioritised drug targets with trial or experimental evidence and targets with robust MR/colocalization evidence of association with COVID-19 severity, we further conducted a phenome-wide MR analysis (MR-PheWAS) to identify potential beneficial and/or adverse effects of these targets on other human diseases. The QTLs for the prioritised targets were chosen as the exposures for the MR-PheWAS. For the outcomes, 49 viral infection phenotypes from the GWAS Catalog (https://www.ebi.ac.uk/gwas/downloads/summary-statistics), 501 human diseases and 72 disease related traits (e.g. blood lipids) selected from the IEU OpenGWAS database^34^ were selected as outcomes for this analysis (Table S7). These 622 outcomes were selected using the following inclusion criteria:

• The GWAS with the greatest expected statistical power (e.g. largest sample size/number of cases) when multiple GWAS records of the same disease/risk factor were available in the Open GWAS database.
• GWAS with betas, standard errors and effect alleles for all tested variants (i.e. full GWAS summary statistics available)

#### Effects of alternative splicing of the prioritised targets on COVID-19 severity

For the six prioritised proteins, we conducted tissue-specific MR and colocalization of splicing events on COVID-19 severity using sQTL data from GTEX v8.^35^ After selection based on *P* value cut-off (*P* < 5 × 10^−8^) and LD clumping (r^2^<0.01), 13 splicing quantitative trait loci (sQTLs) of four genes (*ENTPD5, ICAM1, OAS1* and *SERPINA1*) in six tissues were selected as instruments for the MR. The HGI v6 and GenOMICC v2 COVID-19 severity data in Europeans were used as outcomes. The Wald ratio test (since only one variant was available for each splicing event), and genetic colocalization were conducted to estimate the putative causal roles of splicing events on COVID-19 severity.

#### Analysis software

The MR analyses (including Wald ratio and IVW) were conducted using the TwoSampleMR R package (github.com/MRCIEU/TwoSampleMR).^36^ The colocalization analysis was conducted using the “coloc” and “moloc” packages.^31^^,^^33^ The MR results were plotted as Manhattan plots and forest plots using code derived from the ggplot2 package in R (https://cran.r-project.org/web/packages/ggplot2/index. html).

### Ethics

No ethical approval was required for the present study, since all analyses were only based on publicly available summary statistics without accessing individual-level data. The included GWAS studies all received informed consent from the study participants and have been approved by pertinent local ethical review boards.

## Results

### Estimated causal effects of targets on COVID-19 severity

For the main MR analysis in European ancestry, the genetically-predicted protein level of ENTPD5 showed a positive effect on COVID-19 severity after multiple testing correction (odds ratio [OR] of COVID-19 severity per standard deviation change of protein level= 2.07, 95%CI=1.47 to 2.92, *P* = 3.29 × 10^−5^; Table S8A) using data from HGI. The genetically-predicted protein levels of OAS1 (OR=0.440, 95%CI=0.315 to 0.615, *P* = 1.57 × 10^−6^), FCRL3 (OR=1.032, 95%CI=1.030 to 1.034, *P* = 2.40 × 10^−191^) and ICAM5 (OR=0.780, 95%CI=0.691 to 0.880, *P* = 5.74 × 10^−6^) showed MR association with COVID-19 severity (Table S8B) using data from GENOMICC. As an example, genetically-predicted IL6R showed a marginally protective effect on COVID-19 severity in European ancestry (OR=0.890, 95%CI=0.827 to 0.970, *P* = 2.84 × 10^−3^). The precision of effect estimate was weaker in African ancestry but with little evidence of heterogeneity with the European effect (OR=0.925, 95%CI=0.729 to 1.174, *P* = 0.522; P for pair-wise Z-score test=0.763; Table S8C). For the pQTL instruments of IL6R, the alleles associated with higher soluble protein levels of the protein have been shown to lead to lower intracellular pathway activation,^37^ indicating consistency of direction with the therapeutic approach of IL6 inhibitor on COVID-19.^3^

For the MR analysis on transcripts, expression levels of two genes, *LZTFL1* and *SLC4A10*, showed robust MR evidence on COVID-19 severity using data from GenOMICC (Table S9A). However, none of the genes with genetically-predicted expression data showed robust MR evidence using data from HGI (Table S9B).

**Figure 2:**
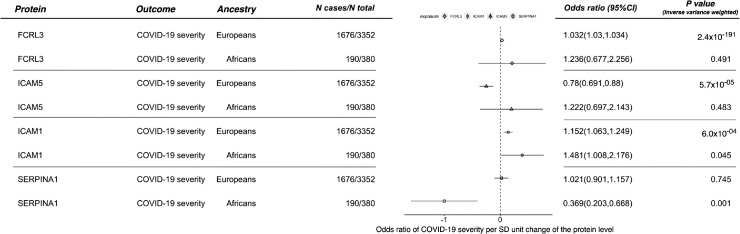
**Mendelian randomization estimates of circulating level of four protein targets on COVID-19 severity in European and African ancestries.** Note: *P* value refers to the inverse variance weighted Mendelian randomization *P* value.

For the main MR analysis in African ancestry, the genetically-predicted protein level of SERPINA1 showed a robust effect on COVID-19 severity in African ancestry (OR=0.369, 95%CI=0.203 to 0.668, *P* = 9.96 × 10^−4^), but showed little evidence of an effect in European ancestry (OR=1.021, 95%CI=0.901 to 1.157, *P* = 0.745; Figure 2). Using a more relaxed *P*-value threshold of 0.05, we found that expression level of one additional protein, ICAM1, showed marginal effect on COVID-19 severity in European ancestry (OR=1.136, 95%CI=1.059 to 1.218, *P* = 3.57 × 10^−4^; Table S8B) and in African ancestry (OR=1.481, 95%CI=1.008 to 2.176, *P* = 0.045; Table S8D).

To validate the top findings, we conducted MR of the prioritised proteins using data from HGI v6 and GenOMICC v2. Protein level of OAS1 showed an effect on COVID-19 severity in Europeans using both HGI v6 (OR=0.827, 95%CI=0.732 to 0.934, *P* = 2.3 × 10^−3^) and GenOMICC v2 data (OR=0.735, 95%CI=0.627 to 0.863, *P* = 1.7 × 10^−4^), where no valid OAS instrument was available in Africans. ICAM1 showed strong effect on COVID-19 severity in Europeans (OR=1.113, 95%CI=1.075 to 1.152, *P* = 1.5 × 10^−9^) and a suggestive effect in Africans (OR=1.275, 95%CI=1.031 to 1.575, *P* = 0.025) using GenOMICC v2 data, with little evidence of heterogeneity of the effect estimates across ancestries (P of pair-wise Z-score test=0.215; Table S8E). SERPINA1 showed an effect on COVID-19 severity in African ancestry using the GenOMICC v2 data (OR=0.481, 95%CI=0.345 to 0.673, *P* = 1.9 × 10^−5^), where the heterogeneity test suggested a distinct effect estimate of SERPINA1 across the two ancestries (P of pair-wise Z-score test=7.70 × 10^−6^; Table S8E). The SERPINA1 instrument in African ancestry, rs6647, is almost in no LD with the three SERPINA1 instruments, rs112209366, rs17580 and rs28929474 (LD r^2^<0.1 in the 1000 Genome Europeans and Africans). These results replicated findings in the main analysis. However, ENTPD5, ICAM5 and FCRL3 did not replicate well using the HGI v6 outcome data (Table S8E). Whether they can be prioritised as drug targets needs further validation.

For the 353 prioritised targets with experimental or trial evidence, all of them showed little MR evidence of a causal effect on COVID-19 severity. Only expression of 35 genes and one protein (NPC2) showed nominal association on COVID-19 severity (we used a lenient *P* value threshold of P < 0.05, maximizing the number of possible genes analysed but also allowing readers to filter out associations should they wish to apply a more stringent threshold, Table S10A and S10B). These 36 targets were human proteins that interact with SARS-CoV-2 proteins^10^ (Table S4).

**Figure 3:**
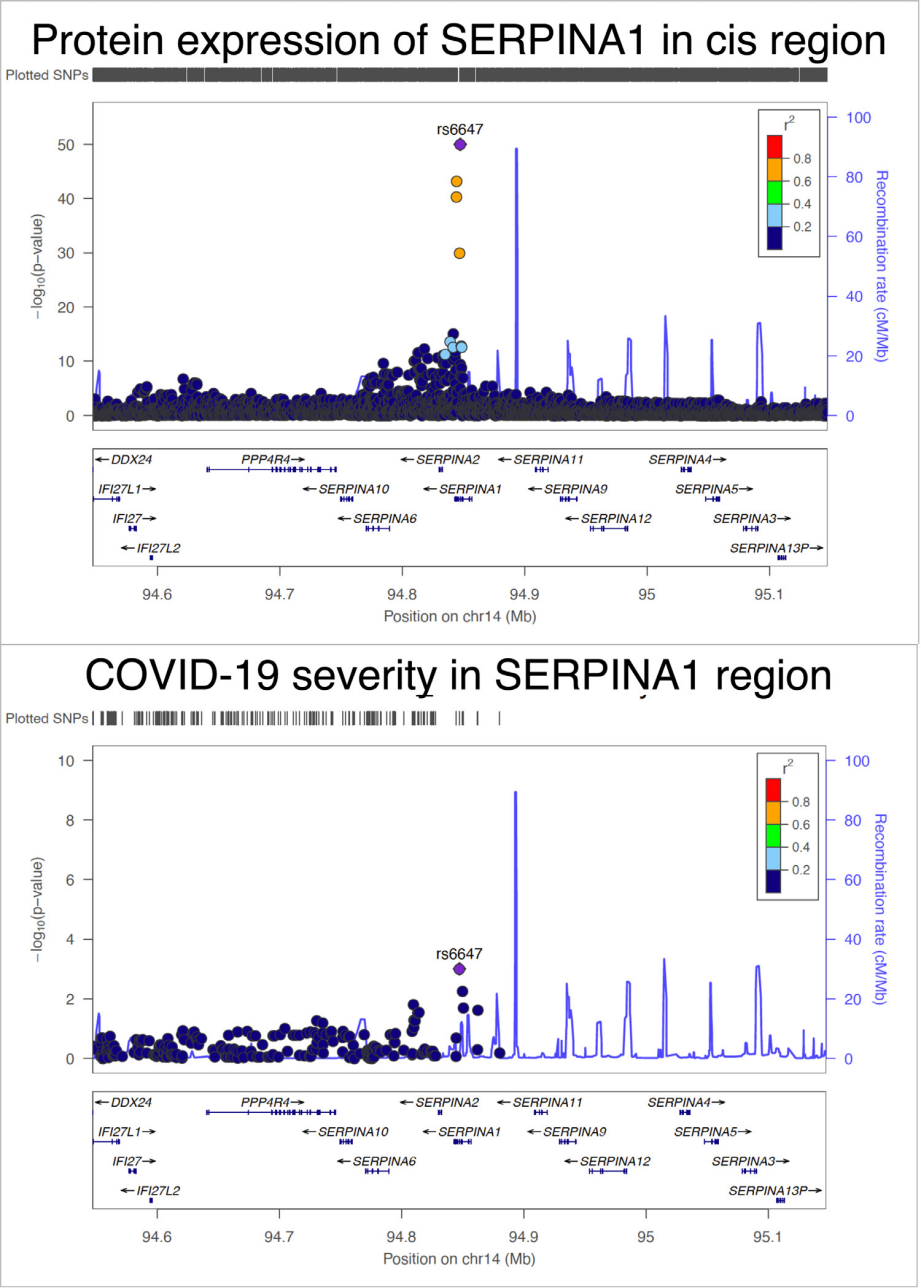
**Regional association plot of protein expression of SERPINA1 and COVID-19 severity in the *SERPINA1* region in African ancestry.** The two regional plots refer to the genetic associations of (A) protein expression of SERPINA1; (B) and COVID-19 severity. The X-axis is the chromosome and position of the *SERPINA1* region.

For the six targets, with strong MR evidence of a causal effect on COVID-19 severity, we conducted single-trait colocalization to distinguish causality from confounding by LD. This analysis showed evidence of a shared genetic effect between protein levels of ENTPD5 (colocalization posterior probability [PP]=99.9%), OAS1 (PP=95.8%), FCRL3 (PP=100%), ICAM5 (PP=78.1%) and ICAM1 (PP=91.8%) with COVID-19 severity in European ancestry, and weaker evidence of a shared effect of protein level of SERPINA1 with COVID-19 severity in African ancestry (PP=64.3%) (Figure 3 and Table S11A).

### Shared causal effect between gene expression and protein level of prioritised drug targets and COVID-19 severity

**Figure 4:**
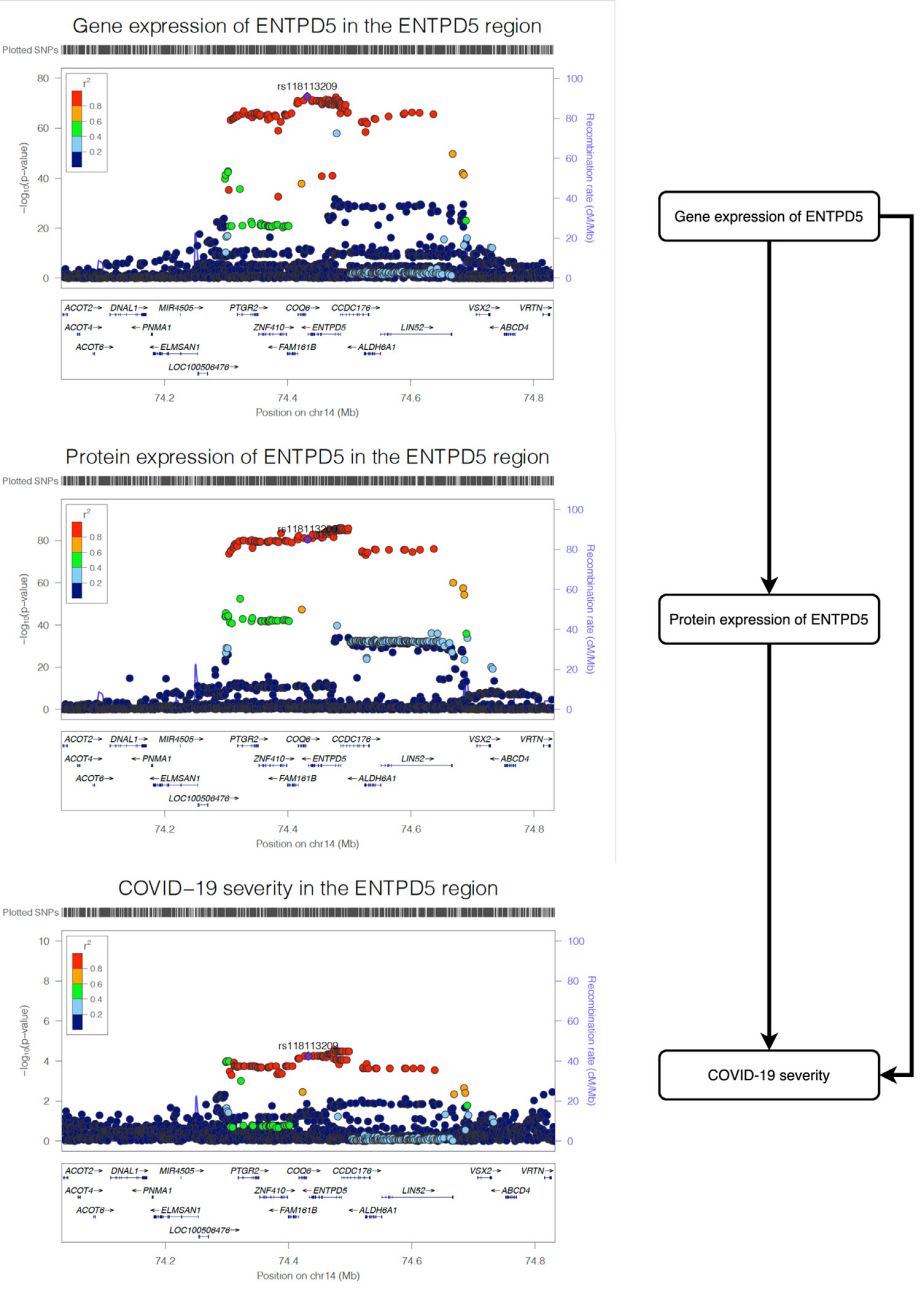
**Regional association plot of gene expression of *ENTPD5*, protein expression of ENTPD5 and COVID-19 severity in the *ENTPD5* region in European ancestry.** The three regional plots refer to the genetic associations of (A) gene expression of *ENTPD5*; (B) protein expression of ENTPD5; (C) and COVID-19 severity. The X-axis is the chromosome and position of the *ENTPD5* region.

We observed strong MR (*P* = 5.66 × 10^−5^) and colocalization (PP=99.9%) evidence to support the effect of gene expression level of *ENTPD5* on COVID-19 severity (Table S11B). The multi-trait colocalization analysis suggested robust colocalization evidence to support shared genetic aetiology of the three traits (shared probability=95.4%; Figure 4; Table S11C), which further strengthens the evidence level of the association between ENTPD5 and COVID-19 severity. For OAS1, we observed little evidence of a causal effect of gene expression level on COVID-19 severity (*P* = 0.568) (Table S11B).

### Estimated beneficial and adverse effects of the prioritised targets on other complex diseases

We estimated the potential beneficial and/or adverse effects of the protein and gene expression levels of the prioritised targets on 622 traits (Table S7) using MR-PheWAS. 45,938 target-disease associations were tested in plasma proteome and/or transcriptome in whole blood (*P* < 1.09 × 10^−6^ at a Bonferroni-corrected threshold). Where data were available, we also tested the tissue-specific effects of gene expression of the same targets on the outcome phenotypes using data from GTEx. Overall, 372,830 target-disease associations were estimated in the 11 COVID-19 relevant tissues.

For the six prioritised drug targets, several additional beneficial effects of these targets (in addition to their effects on COVID-19 severity) were observed, which included inhibition of FCRL3 level on reducing rheumatoid arthritis, lymphoid leukaemia, hypothyroidism and hyperthyroidism; increased OAS1 level on reducing Alzheimer's disease risk; and inhibition of ICAM1 level on reducing risk of neo-extraversion, hypertension and skin cancer and reducing body mass index and diastolic blood pressure level (Table S12A).

For adverse effect identification, the MR-PheWAS suggested that increased level of SERPINA1 increased systolic and diastolic blood pressure level; and inhibition of ICAM1 increased the risk of irritability, allergy, and nervous feelings. However, the effect sizes were marginal (OR=1.01 to 1.02) and not comparable to the beneficial effect on COVID-19 (OR=0.88). Increased ICAM5 level increased risk of inflammatory bowel disease (Table S12A). ENTPD5, OAS1 and FCRL3 showed little effects on other diseases (Table S12A and Figure S2).

For the 353 targets prioritised by literature evidence, we observed 833 target-disease associations with robust MR evidence in the 11 tested tissues. Using the same omics data as the MR analysis, 726 of the 833 (87.2%) associations also showed strong colocalization evidence (colocalization probability > 70%) (Table S12B).

### Estimated causal effects of alternative splicing events of the prioritised targets on COVID-19 severity

For the six prioritised drug targets, we identified 13 sQTLs associated with splicing events of four of the targets (Table S13A). The MR analyses using these sQTLs as instruments suggested that alternative splicing events of the *OAS1* and *ICAM1* in some tissues (including adipose, adrenal gland and artery tissues) showed marginal effects on COVID-19 severity (MR *P* value<1 × 10^−3^). The colocalization analysis further suggested that splicing events of *OAS1* and *ICAM1* in adipose tissues showed some evidence of colocalization with COVID-19 severity (colocalization probability=92%∼98%; Table S13B), which further strengthens the putative causal role of OAS1 and ICAM1 on COVID-19 severity via alternative splicing events.

## Discussion

The genetic study of molecular traits (omics) provides a cost-effective approach to prioritise drug targets and predict drug response in multiple ancestries.^13^^,^^17^ In this study, we applied omics MR in multiple ancestries to prioritise drug targets for COVID-19 severity. Our omics MR and colocalization analysis of over 400K target-disease pairs identified putative causal effect of levels of six proteins on COVID-19 severity, where four of them, ENTPD5, OAS1, FCRL3, ICAM5, showed evidence of effects in European ancestry; SERPINA1, showed evidence of an effect in African ancestry, and ICAM1 showed evidence of a bi-ancestry effect. The MR-PheWAS of these prioritised targets showed little evidence of effect on other complex traits, which implies that these targets are unlikely to have a major adverse effect on complex human diseases. To enable the evidence to be widely accessible for COVID-19 drug target research, we released all our MR results via the EpiGraphDB platform (http://epigraphdb.org/covid-19/ctda/).

The *SERPINA1* gene is a serine protease inhibitor, alpha-1 antitrypsin, that may protect against COVID-19 by inhibiting TMPRSS2. Several clinical trials of SERPINA1 have been initiated for COVID-19 treatment in Saudi Arabia (NCT04385836), Spain (NCT04495101), the USA (NCT04547140), and Ireland (EudraCT 2020-001391-15). However, no ancestry information had been provided for these ongoing trials. In this study, our results provide genetic evidence to suggest that increased SERPINA1 level reduced risk of COVID-19 severity in African ancestry. One recent epidemiology study further suggested that alpha-1 antitrypsin deficiency alleles PiZ and PiS (rs28929474 and rs17580 respectively) may contribute to national differences in COVID-19 infection, severity, and mortality rates.^38^ However, several recent studies, suggested that urgent action is needed for this target, as alpha1-antitrypsin deficiency could be a risk factor for COVID-19.^39^^,^^40^ Whether SERPINA1/alpha1-antitrypsin can be considered as a drug target for COVID-19 needs investigation in future animal studies and clinical trials.

Two genes, ICAM1 and ICAM5, belonging to the intercellular adhesion molecule family, showed effects on COVID-19 severity in our study. Proteins encoded by these two genes bind to the leukocyte adhesion LFA-1 protein, which is related to immune response. A recent study suggested that ICAM5/TYK2 gene is associated with severe COVID19.^41^ Another study further suggested ICAM1 as a potential prognostic indicator for COVD-19 infection.^42^ Our results support these findings suggesting further investigation of the role of the ICAM gene family is merited.

The *ENTPD5* gene encodes the NTPase Ectonucleoside Triphosphate Diphosphohydrolase 5, an enzyme mediating extracellular catabolism. A specific role of ENTPD5 is to promote host protein N-glycosylation for proper protein folding (information from Open Targets). SARS-CoV-2 spike protein is extensively N-glycosylated and blocking viral N-glycan biosynthesis was shown to inhibit viral entry.^43^ Our results suggested that both gene and protein expression levels of *ENTPD5* showed an effect on COVID-19 severity, which provides evidence to support the examination of the direct role of ENTPD5 inhibition on SARS-CoV-2 viral entry and, subsequently, in the prevention of severe COVID-19 in future studies.

This study identified six protein targets that may prevent COVID-19 severity in two ancestries separately. Our results suggested that ICAM1 showed putative causal effects on COVID-19 severity in both ancestries, with splicing events potentially mediating the effect. The same target may benefit body weight control. Whether this target can be considered as a target for COVID-19 severity needs further validation in future clinical trials. The beneficial effect of increased SERPINA1 on COVID-19, especially in Africans, is worth careful investigation in future studies. OAS1 and three other proteins showed putative causal evidence in European ancestry (but with weaker effects in Africans). The OAS1 effect on COVID-19 in Europeans has been well-replicated in previous GWAS and MR studies,^21^ with our findings also validating the effects of OAS1 on COVID-19 severity via alternative splicing.^44^ However, there is no good instrument in Africans yet. Whether this effect can be extended to other ancestries needs further investigation.

### Caveats and limitations

Some limitations of our analysis are worth noting. Whilst initiatives are underway to collect genetic information for COVID-19 patients (e.g. the COVID-19 host genetics initiative, https://covid-19genehostinitiative.net/), the recent initial GWASs of COVID-19 found several genetic association signals in Europeans, but COVID-19 data in other ancestries are still lacking, which highlights the importance of well-powered genetic studies in non-European ancestries. Given the sample size difference between the European data and African data, we considered the European MR top findings as European prioritised drug targets rather than European-only findings. Second, recent MR studies of COVID-19 identified a few gene targets associated with COVID-19,^18^, ^19^, ^20^, ^21^, ^22^ but these studies downplayed consideration of potential biases in the data. For example, if we use a mixture of population samples as controls (which includes unexposed individuals and asymptomatic and untested cases), we will not be able to distinguish the causal effects of being exposed to SARS-CoV2, infected by the virus, or progressing to severe COVID-19 after infection.^26^ A GWAS using an unbiased population sample screened to detect infection by SARS-Cov-2 (e.g. from antibody tests) will be helpful to disentangle this issue, but such a GWAS does not yet exist. Our study excluded the five available COVID-19 GWAS datasets (with populations or self-reported data as controls) to minimise the influence of mixed controls. We only used hospitalised COVID-19 vs. non-hospitalised COVID-19 as the outcome for MR, which had the most reliable definition of cases and controls. However, given the GWAS of hospitalized COVID-19 vs. not hospitalized COVID-19 was still selected on the basis of COVID-19 status, collider bias could still be an issue (see Figure S3). To identify potential colliders that will bias the MR estimates, we conducted MR-PheWAS of the six prioritised targets. These targets did not show much evidence of a causal effect on major diseases (e.g. asthma), which suggested that protein level change of these targets are not obviously acting on selection via an effect on a disease which increases likelihood of COVID-19 testing. However, the targets prioritised by our study should still be carefully reviewed in future trials before clinical application. Third, the drug targets evaluated in this study were proxied using a limited number of instruments, which means the putative causal effects rely on a few genetic instruments. Thus, these associations support causality but do not prove it, as horizontal pleiotropy remains an alternative possibility. In addition, some of the pQTL instruments are in weak to modest LD to each other. These were included in the MR model, but LD was addressed by use of the generalized IVW method to estimate MR effect sizes. This method includes a LD matrix in the IVW model to control for the LD among instruments, which results in the inclusion of more instruments in the MR model and may boost power of the MR findings. Fourth, even though our results suggest some biological links between the target and diseases, these only provide evidence for the very first step of the drug development process. Fifth, whilst these are plausible targets for COVID-19 severity, we cannot predict whether successful intervention would impact on risk of infection or other disease characteristics relevant to public health (e.g. viral shedding). Finally, the genetic colocalization methods are increasingly being used together with MR to inform drug development.^45^ Recent colocalization methods have started to deal with multiple signals in the same genomic region,^46^^,^^47^ which overcomes a major limitation of the method. However, we need to interpret colocalization results with care. In particular, for genomic regions with long LD blocks or a complex LD structure and GWAS data with limited power, colocalization yield unreliable results.

In conclusion, this study prioritised six proteins as potential drug targets for COVID-19 severity using an integrated genetic approach. Five proteins showed ancestry-specific effects, which evidences the value of genetic approaches in predicting drug response in different populations. This provides the very first step towards evaluating intervention targets that are worth following-up for all types of coronaviruses.

## Contributors

J.Z. and Y.M.Z. selected the drug targets; J.Z. and H.L.Z. performed the Mendelian randomization analysis; J.Z., H.L.Z. and D.B. performed the colocalization analysis; J.Z. conducted the triangulation between MR and drug trials; J.Z. and Y.M.Z. conducted the drug target prioritization; H.L.Z. conducted the MR analysis of splicing events. Y.L. developed the database and web browser; J.Z. and Y.M.Z. wrote the manuscript; D.B., Y.L., L.W., X.Z.L., H.Z. and T.R.G. reviewed the paper and provided key comments; J.Z., G.D.S., and T.R.G. conceived and designed the study and oversaw all analyses. All authors read and approved the manuscript.

## Data sharing statement

We have made all MR results openly available to browse or download at the COVID-19 Target-Disease Atlas (CTDA) browser within the EpiGraphDB platform (http://epigraphdb.org/covid-19/ctda/). This includes 14,873 unique target-COVID-19 severity associations evidence for the omics MR as well as 372,830 unique target-disease associations evidence for 353 targets on 622 diseases/phenotypes in 11 SARS-CoV-2 related tissues. Users are able to query the study results by the targeted gene/protein name and QTL SNPs via the online platform, and the results are presented in searchable tables as well as volcano plots. In addition, users can programmatically access the results using the /covid-19/ctda endpoints in the application programming interface (API) of EpiGraphDB via http://api.epigraphdb.org/.

To support reproducibility of our pipeline, we have made a Github repository to share the scripts for running Mendelian randomization and follow-up analyses.

https://github.com/MRCIEU/epigraphdb-ctda.

## Declaration of interests

No competing interests.

## Appendix Supplementary materials

Image, application 1

Image, application 2

Image, application 3
