# Supplementary material for: Multi-ancestry Mendelian randomization of omics traits revealing drug targets of COVID-19 severity
Source: eBioMedicine. 2022 Jun 27;81:104112. doi: 10.1016/j.ebiom.2022.104112 (PMC9235320; doi:10.1016/j.ebiom.2022.104112)
Supplement: Supplementary file 1 [file mmc1.docx]

## References in Supplementary documents

1 Sun BB, Maranville JC, Peters JE, *et al.* Genomic atlas of the human plasma proteome. *Nature* 2018; **558**: 73–9.

2 Folkersen L, Fauman E, Sabater-Lleal M, *et al.* Mapping of 79 loci for 83 plasma protein biomarkers in cardiovascular disease. *PLoS Genet* 2017; **13**: e1006706.

3 Suhre K, Arnold M, Bhagwat AM, *et al.* Connecting genetic risk to disease end points through the human blood plasma proteome. *Nat Commun* 2017; **8**: 14357.

4 Yao C, Chen G, Song C, *et al.* Genome-wide mapping of plasma protein QTLs identifies putatively causal genes and pathways for cardiovascular disease. *Nat Commun* 2018; **9**: 3268.

5 Emilsson V, Ilkov M, Lamb JR, *et al.* Co-regulatory networks of human serum proteins link genetics to disease. *Science* 2018; Aug 2.

6 Zheng J, Haberland V, Baird D, *et al.* Phenome-wide Mendelian randomization mapping the influence of the plasma proteome on complex diseases. *Nat Genet* 2020; **52**: 1122–31.

7 Võsa U, Claringbould A, Westra HJ, Bonder MJ, Deelen P, Zeng B, et al. Large-scale cis- and trans-eQTL analyses identify thousands of genetic loci and polygenic scores that regulate blood gene expression. Nat Genet. 2021 Sep;53(9):1300-1310.

8 Zhao H, Rasheed H, Nøst TH, *et al.* Proteome-wide Mendelian randomization in global biobank meta-analysis reveals multi-ancestry drug targets for common diseases. *MedRxiv* 2022; 2022.01.09.21268473.

9 Cotto KC, Wagner AH, Feng Y-Y, *et al.* DGIdb 3.0: a redesign and expansion of the drug-gene interaction database. *Nucleic Acids Res* 2018; **46**: D1068–73.

10 Mendez D, Gaulton A, Bento AP, *et al.* ChEMBL: towards direct deposition of bioassay data. *Nucleic Acids Res* 2019; **47**: D930–40.

11 Gordon DE, Jang GM, Bouhaddou M, *et al.* A SARS-CoV-2 protein interaction map reveals targets for drug repurposing. *Nature* 2020; April 30.

12 Gralinski LE, Ferris MT, Aylor DL, *et al.* Genome Wide Identification of SARS-CoV Susceptibility Loci Using the Collaborative Cross. *PLoS Genet* 2015; **11**: e1005504.

13 Aguet F, Barbeira AN, Bonazzola R, *et al.* The GTEx Consortium atlas of genetic regulatory effects across human tissues. BioRxiv. 2019; Oct 3.

14 Gillies CE, Putler R, Menon R, *et al.* An eQTL Landscape of Kidney Tissue in Human Nephrotic Syndrome. *Am J Hum Genet* 2018; **103**: 232–44.

15 Koscielny G, An P, Carvalho-Silva D, *et al.* Open Targets: a platform for therapeutic target identification and validation. *Nucleic Acids Res* 2017; **45**: D985–94.

16 Wishart DS, Knox C, Guo AC, *et al.* DrugBank: a comprehensive resource for in silico drug discovery and exploration. *Nucleic Acids Res* 2006; **34**: D668-72.

17 Finan C, Gaulton A, Kruger FA, *et al.* The druggable genome and support for target identification and validation in drug development. *Sci Transl Med* 2017; **9**.
